# Biomechanical Evaluation of a Novel Ceramic Implant for Canine Cranial Cruciate Ligament Rupture Treatment: A Finite Element Analysis Approach

**DOI:** 10.3390/ani14223296

**Published:** 2024-11-15

**Authors:** Mark Leon Lang, Matthias Lüpke, Maximilian Götz, Holger A. Volk, Jan Klasen, Oliver Harms

**Affiliations:** 1 Small Animal Medicine Clinic, University of Veterinary Medicine Hannover Foundation, 30559 Hannover, Germanyholger.volk@tiho-hannover.de (H.A.V.);; 2 Department for General Radiology and Medical Physics, University of Veterinary Medicine Hannover Foundation, 30173 Hannover, Germany; matthias.luepke@tiho-hannover.de; 3 Small Animal Clinic Germersheim, 76726 Germersheim, Germany; 4 Specialist Center for Small Animal Medicine, 30885 Langenhagen, Germany

**Keywords:** ceramic implant, tibial tuberosity advancement, finite element analysis, cranial cruciate ligament rupture, biomechanics, canine orthopaedics

## Abstract

This research investigates the biomechanical effects of a novel ceramic implant for the treatment of canine cranial cruciate ligament rupture (CCLR) based on the tibial tuberosity advancement (TTA) method using finite element analysis (FEA). A 3D FEA of the tibiofemoral joint simulating the applied forces (44.5% of body weight) during the mid-stance phase (joint angle 135°) of the dog’s stride was performed. Three conditions were considered for each joint: the physiological condition, the pathological condition with CCLR and the restored condition after TTA. Eight cadavers were used to create fifteen paired knee joints. The results showed significant differences in the forces that could be measured in the patellar tendon (PT) and in the cranial displacement of the tibial tuberosity between the conditions. The PT forces increased in the pathological state and continued to increase in the restored state, while the cranial displacement of the tibial tuberosity increased in the pathological state and decreased again in the restored state. Correlation analyses revealed significant correlations between PT forces, body weight and cranial displacement. The FEA provides initial insights into the force distribution and functionality of the ceramic implant. However, further testing is required to validate reliability and evaluate the efficacy of the implant.

## Simple Summary

The aim of this research was to evaluate the effectiveness of a newly developed ceramic implant for the treatment of cranial cruciate ligament rupture in dogs using finite element analysis. Loading on the knee joint was simulated in different conditions, including the physiological condition, the ruptured cranial cruciate ligament and after implantation of the new implant. Particular attention was paid to the effects of the implant on the biomechanics of the tibiofemoral joint. The results showed significant differences between the conditions in terms of the measured forces on key structures, such as the patellar tendon and the menisci, as well as in terms of joint stability. The implant led to dynamic changes in force distribution, suggesting positive effects on joint stability and function. However, further validation is required to ensure reliability and efficacy. This underlines the need to further refine the modelling techniques.

## 1. Introduction

Cranial cruciate ligament rupture (CCLR) is a common orthopaedic condition in dogs [1], with large breeds and overweight, 2–10-year-old and neutered animals being predisposed [2]. This is not solely a traumatic event but is primarily linked to degenerative changes in the cranial cruciate ligament (CCL) characteristic of canine cruciate ligament disease [3]. This condition involves a degenerative progressive ligament weakening and collagen matrix degeneration, which, when advanced, results in CCLR [4]. The intact cranial cruciate ligament (CCL) limits cranial movement of the tibia and inward rotation of the limb [5]. The resulting instability after CCLR leads to secondary OA, which should be treated by surgical restabilisation [6]. Osteotomy procedures have been established, especially in dogs over 30 kg [7], which cause a biomechanical rearrangement of the knee joint and render the CCL superfluous by neutralising the cranially directed tibiofemoral shear forces in various ways [8]. Tibia Plataeu Levelling Osteotomy (TPLO) [9] is based on the assumption that the joint reaction force runs parallel to the longitudinal axis of the tibia and can be brought into parallel alignment with the longitudinal axis by rotating the caudally sloping tibial plateau, which prevents subluxation. In contrast, the tibial tuberosity advancement (TTA) [10] system assumes that the joint reaction force runs almost parallel to the patellar tendon (PT) and is orientated perpendicular to the tibial plateau by a cranial advancement of the tibial tuberosity. To maintain the distance between the plateau and tuberosity, a metal cage is inserted in all TTA variations, which must remain permanently in the osteotomy gap [11]. Due to the inert biological surface of the metal implant, it is necessary to insert an autogenous or allogenic bone graft [12] to ensure optimal bone healing and prevent uneven bone remodelling. Compared to conventional metal implants, biodegradable ceramic implants exhibit better ingrowth behaviour [13,14] and less biofilm formation [15]. This reduced biofilm could be advantageous with regard to wound infections, as the bacteria in infected metal implants have a significant tolerance to antibiotics due to the biofilm, which can lead to an increase in the minimum inhibitory concentration by up to 100 times [16]. In a retrospective study [17] of 1613 dogs, surgical site infection was the most common complication after TTA, with a prevalence of 8.4%, of which 1.5% involved complications so severe that surgical revision was required. In about a third of serious cases, the metal implants have to be removed [18], which invalidates the success of the TTA and can lead to further complications such as the avulsion of the tibial tuberosity. Based on these findings and with the aim of utilising the biocompatibility of the ceramic, we developed a cylindrical ceramic implant that is inserted into a pre-drilled hole along the osteotomy gap, based on an already established surgical technique.

To evaluate the performance, functionality and behaviour of the ceramic implant fully, both a finite element analysis (FEA) and a biomechanical study were carried out on 15 joints. This interdisciplinary approach made it possible to analyse not only the biomechanical aspects but also the potential clinical benefits and challenges of this innovative implant. A detailed 3D FEA model of the TTA system was developed to investigate in detail the mechanical behaviour of the tibiofemoral joint and the forces generated in the middle of the dog’s stride.

## 2. Materials and Methods

The present models are based on eight euthanised dog cadavers selected for reasons unrelated to this study. The following inclusion criteria were considered: included limbs had no pre-reported evidence of orthopaedic disease or hind limb lameness. One hind limb was therefore excluded, which is why 15 paired knee joint models were created from the eight dogs. The specimens were frozen, and before fitting the ceramic implant, these were thawed at room temperature for approximately 24 h. All cadavers were scanned with the Philips IQon Spectral CT (Phillips GmbH Market DACH, 22335 Hamburg, Germany) with a slice thickness of 0.8 mm and an overlap of 0.5 mm before and after fitting the ceramic implant. Three models were created for each joint: condition 1, representing the physiological state; condition 2, representing the pathological state with a torn anterior cruciate ligament, and condition 3, representing the restored state using the TTA method. This resulted in a total of 45 analyses.

The ceramic implant, made of zirconia-toughened alumina ceramic foam, is available in various sizes to accommodate different dog breeds and sizes and is inserted following a technique based on an established surgical procedure. Due to an ongoing patent process, further details cannot be disclosed at this time.

**Figure 1:**
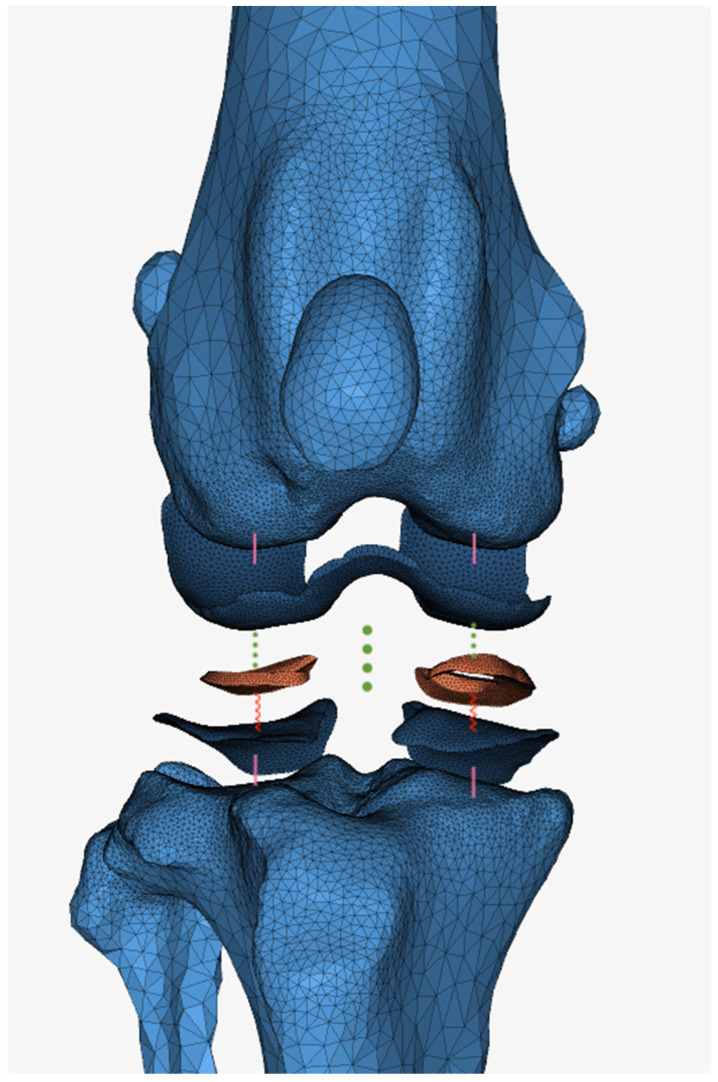
View from cranial into the joint including contact definitions with visual mesh structure. Properties: light blue: bone; dark blue: cartilage; orange: meniscus. Contacts: pink straight connection: tie; red jagged connection: friction; green dotted connection: slide.

The bones and the implant involved in the knee joint were converted into individual STL files by segmenting the CT images (Materialise Mimics Version 22.0) and exported for integration into subsequent computer programs (Materialise 3-Matic Version 17.0). In a subsequent step, the surface meshes of the bones were processed and provided with a tetrahedral TET 10 mesh, which enables a precise representation of the anatomical structures, taking into account their irregularities and withstanding large deformations [19]. This mesh had an edge length of 0.8 mm in the area of the epiphysis close to the knee joint, which was gradually enlarged to 6.4 mm up to the epiphysis far away from the knee joint in order to reduce the number of elements (Figure 1). Due to the insufficient visualisation in the CT, the soft tissue structures, such as menisci and cartilage surfaces, were subsequently created in Materialise 3matic Version 17.0. The articular cartilage was simulated at the corresponding location with a thickness of 0.65 mm [20] and a mesh of 0.6 mm. The menisci were fitted into the joint gap at the corresponding points and provided with a 0.4 mm mesh.

Once the joints had been prepared, they were transferred to a pre-processing program (Altair Hypermesh Version 2022.3) where the boundary conditions for the analysis were defined. This marked the initial step of the FEA, followed by the automatic solution of the previously established system of equations, here utilising Altair Optistruct Version 2022.3. Afterwards, post-processing ensued, presenting the results in the form of numerical values and graphical plots (Altair Hyperview Version 2022.3).

The bones, cartilage, menisci and implant were simulated as solids, which meant that the Poisson’s ratio, Young’s modulus, and density of these materials were required for the calculations. The corresponding parameters were taken from current veterinary and human medical literature and are listed in Table 1. With regard to these materials, a simplification assumption was made that they were isotopically homogeneous materials. Concerning bone, no distinction was made between cortical and cancellous bone; instead, bone was considered entirely cortical bone. Ligaments and muscles were modelled as non-linear springs that only respond to tension and not to compression. This excluded time-dependent material properties, such as viscoelasticity, friction and relaxation, from the calculation. A stiffness of 270 N/mm^2^ was assumed for both cruciate ligaments [21], 300 N/mm^2^ for the patellar ligament [22], 80 N/mm^2^ for the collateral ligaments [23] and 50 N/mm^2^ for the muscles [24]. The analysis included the gastrocnemius muscle, while the quadriceps muscle was not simulated to allow for a higher error tolerance. Instead, the patella was fixed to the femur at a distance equal to the physiological length of the patellar ligament [25].

The individual articular cartilage was firmly anchored to the bone using “tie” contacts. The menisci were fixed to the tibial articular cartilage with a comparatively high coefficient of friction, as they are physiologically connected to the tibia via four ligaments. The medial meniscus was assigned a friction coefficient of 0.9, as it is less mobile than the lateral meniscus, for which a coefficient of 0.8 was selected due to its connection with the medial collateral ligament. The implant was connected to the tibia using “Contact Freeze”, as was the contact of the patella with the femur. Frictionless sliding contact was selected for the femoral cartilage and the tibial cartilage as well as for the femoral cartilage and the menisci. This is illustrated in Figure 1.

**Figure A1:**
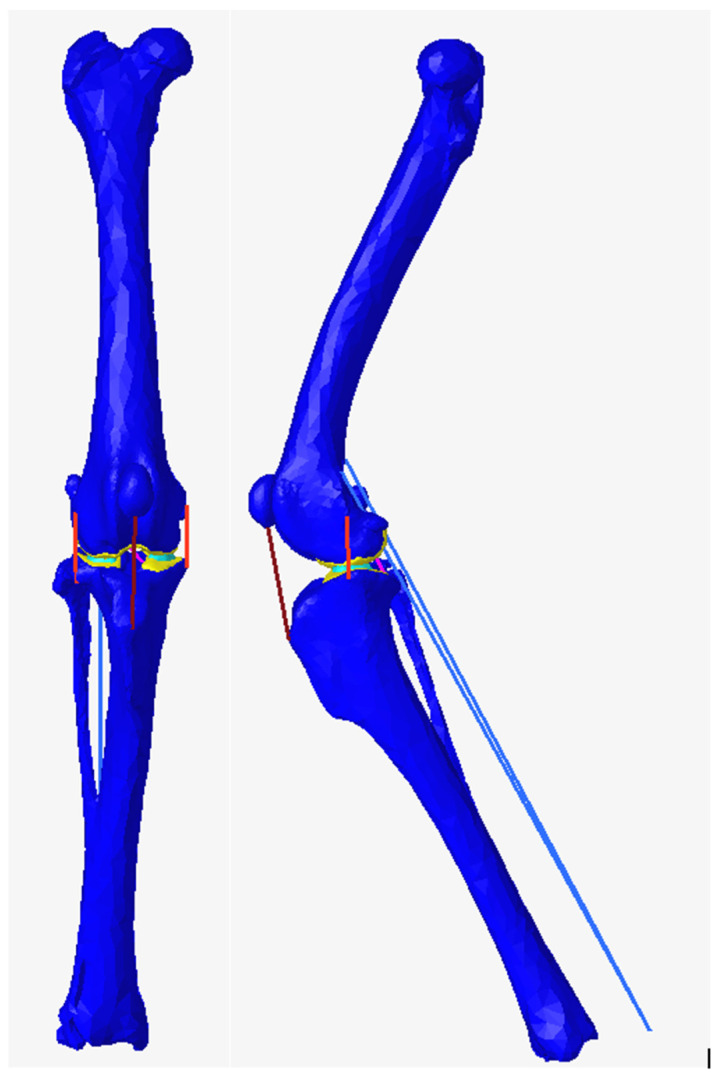
Knee joint with muscles and ligaments in cranial (**left**) and medial (**right**) view; Solids: blue: bone; yellow: cartilage; cyan: menisci. Ligament structures: light red: collateral ligaments; dark red: patellar tendon: pink: cruciate ligaments; blue: gastrocnemius muscle.

The joint was positioned at an angle of 135°, which corresponds to the mid-stance phase of the step [31,32] while walking and the standing angle [33]. The force applied to the hind limb during the stepping phase was 44.5% of body weight (BW) [34], equivalent to 4.37 N/kg, based on a gravitational pull of 9.81 N/kg. This force was applied evenly in 10 steps to the distal tibia. The femur was firmly fixed to the Caput ossis femoris and the insertion of the gastrocnemius muscle. The tibia was restricted in its medial and lateral movement but was free to move in the cranial–dorsal and proximal–distal axes. An image of the knee model including the soft tissue structures can be found in Figure A1 Appendix A.

The finite element analysis (FEA) provides a large amount of calculated data in both graphical and numerical form. This includes stress distributions and compressive loads of the individual TET 10 elements, displacements of the components, and the forces acting on the individual ligaments at the end of the simulation. In this study, various parameters were extracted from these data using Altair Hyperview Version 2022.3, including the pressure distribution in megapascals (MPa) in graphical form, the force distribution in Newtons on the individual ligament structures, and the displacement of the tibial tuberosity in millimetres.

The available data, consisting of 45 collected observations, were analysed using commercially available programs (SAS Enterprise Guide 8.3). The distribution of PT force and tibial tuberosity advancement was subjected to a graphical normal distribution test, whereby the convergence criteria were met. Due to the quantitative nature of the analysed data and the qualitative paired factor of the condition on the values, a mixed model was applied. A mixed model analysis—in particular, the Type 3 Fixed Effects test—was performed to investigate the influence of the condition. The Tukey–Kramer procedure was selected as the “post hoc test”. In addition, a separate correlation analysis was performed for each condition using Spearman’s correlation coefficient. The parameters analysed included the advancement with BW, the force in the patellar ligament with BW, the force in the patellar ligament with advancement, the force in the CCL with BW (condition 1 only) and the force in the CCL with advancement (condition 1 only).

## 3. Results

### 3.1. Canine Specimens

The cadavers used in this study were of different breeds and weights, including one German Shepherd, one Labrador Retriever, one Corgi, one Jack Russell Terrier, one Small Munsterlander, one Cane Corso Italiano and two mixed breeds. The average age at the time of the scans was 7.26 years (standard deviation 4.29 years), and the weight was 22.74 kg (standard deviation 13.11 kg). The dogs were divided into two males, three females and three neutered males.

### 3.2. Strain on Ligaments

**Figure 2:**
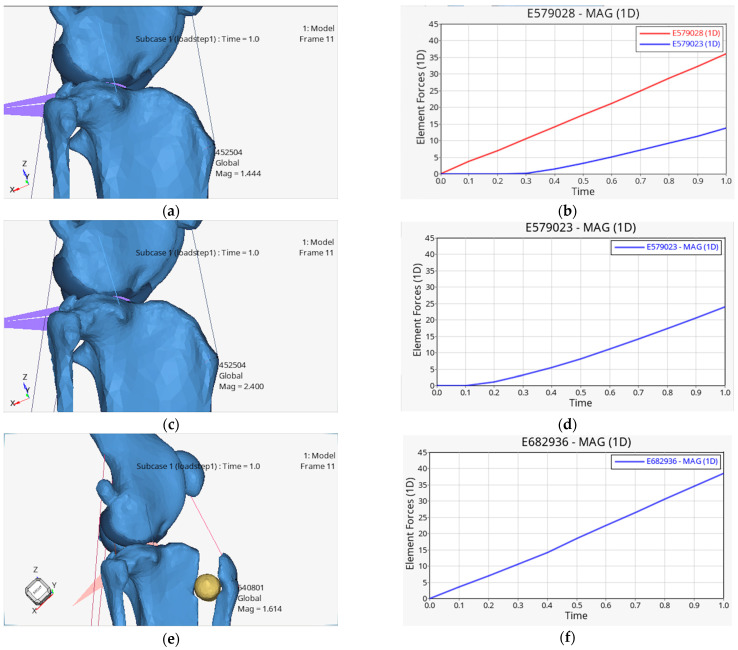
Insight into the post-processing with calculations of the tibial advancement in mm (**a**,**c**,**e**) and the forces on the PT and the CCL (**b**,**d**,**f**). On the x-axis, time steps from 0 to 1 s are displayed, and on the y-axis, force in Newtons is shown. The forces in the CCL are shown in red in b, while the forces in the PT are shown in blue. (**a**,**b**) represent the values of condition 1, (**c**,**d**) the values of condition 2 and (**e**,**f**) the values of condition 3 (condition 1 = physiological, condition 2 = pathological, condition 3 = post-operative).

The results of the calculated loads for the PT were determined in a total of 45 analyses, while results for the CCL were only available in the first condition, as it was not simulated in the other two conditions. The values were recorded at the end of the load during the stepping phase of the stride and are listed in Table 2, both in absolute figures and as a percentage of the force applied in each case. The dynamic increase in load can be seen in Figure 2, using the example of the right limb of dog 1 for all three conditions. The force transmission to the CCL averaged 25% ± 9.1%. The data for the PT showed an increase in the forces in the three conditions, with the force in the PT in condition 1 amounting to 10% ± 7.7%. After removal of the CCL in condition 2, the force transmission to the PT increased to 18% ± 11.6% and increased further to 25% ± 10.4% after the operation.

The calculated force in the PT showed a normal distribution with respect to the three conditions, and a statistically significant difference between the conditions was found using a Type 3 Fixed Effects test with a *p*-value less than 0.05 (*p* = 0.0391). Differential least squares revealed a statistically significant difference between the measured forces in condition 1 and condition 3 with an adjusted *p*-value of 0.0303.

Due to the positive, linear relationship in the scatter plots, the Pearson correlation coefficient was used to test the parameters for correlation. In all three conditions, the force in the PT correlated with both the BW and the advancement. Similarly, the force in the CCL correlated with the BW and the advancement. The individual correlation coefficients with the corresponding *p*-values are listed for each condition in Table A1 Appendix A.

### 3.3. Advancement of the Tibial Tuberosity

Table 3 contains the absolute values as well as percentage changes compared to the advancement in condition 1 and the percentage reduction in advancement after the operation.

The advancement peaks in condition 2 increased on average by 55% ± 31.5% compared to physiological condition 1 and dropped to an increase of 21% ± 21.1% in condition 3, which corresponds to a reduction of −19% ± 7.5% due to the surgery. These changes in advancement contrasted with the calculated forces in the PT, which were highest in condition 3.

The advancement also showed a normal distribution in the three conditions, and a statistically significant difference was found between the conditions (Type 3 Fixed Effects test) with a *p*-value below 0.05 (*p* = 0.0347). Least mean squares analysis showed a statistically significant difference in advancement between condition 1 and condition 2, with a *p*-value of 0.0281 after adjustment. In addition to the aforementioned correlation of the advancement in the individual conditions with the force in the patellar ligament, the advancement in the three conditions also correlated positively linearly with the BW. The Pearson correlation coefficient was 0.92470 (*p*-value <0.0001) for condition 1, 0.85188 (*p*-value <0.0001) for condition 2 and 0.80143 (*p*-value 0.0003) for condition 3.

### 3.4. Pressure Maps

**Figure A2:**
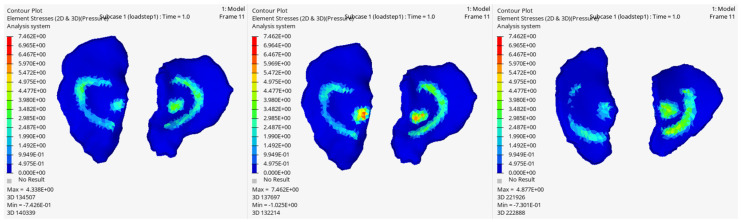
Force distribution in the left leg of dog 3: view of the tibial cartilage from above in the proximal direction. Conditions 1 to 3 are arranged from left to right, with the medial side on the left and the cranial side at the bottom.

**Figure A3:**
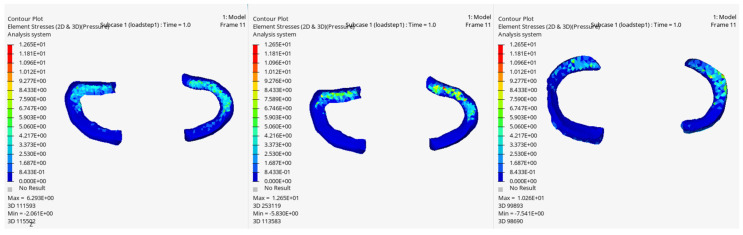
Force distribution in the left leg of dog 3: view of the meniscus from above in the proximal direction. Conditions 1 to 3 are arranged from left to right, with the medial side on the left and the cranial side at the bottom.

**Figure A4:**
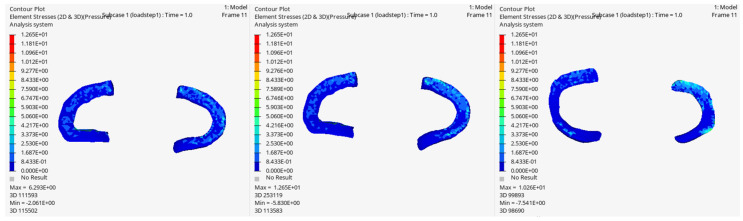
Force distribution in the left leg of dog 3: view of the meniscus from below in the distal direction. Conditions 1 to 3 are arranged from left to right, with the medial side on the left and the dorsal side at the bottom.

**Figure A5:**
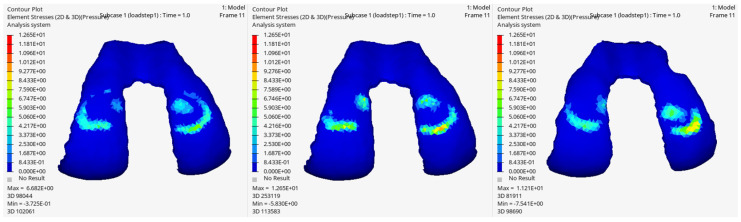
Force distribution in the left leg of dog 3: view of the femoral cartilage from below in the distal direction. Conditions 1 to 3 are arranged from left to right, with the medial side on the left and the dorsal side at the bottom.

The effect of force on bone and cartilage structures was graphically represented by the 15-level coloured scaling of the surface pressure in megapascals. The analysis of the force maps proved to be challenging, as no clear, objective trend was easily recognisable. However, it became apparent that most of the load peaks on the individual structures were recorded in condition 2. This was particularly true for the menisci, where in 13 out of 15 legs, the load peaks in condition 2 were higher than in conditions 1 and 3, where this was the case only once each. The force effect on the implants was also shown graphically, whereby two opposing areas became particularly clear, which corresponded to the contact surface at the insertion site on the bone. The force maps of the right limb of dog 3 can be found in the Figure A2, Figure A3, Figure A4 and Figure A5 as an example.

## 4. Discussion

FEA has established itself as a proven method for investigating the biomechanical functioning of joint pathologies in all areas of medicine and plays an important role in the evaluation of new implants [35,36]. The potential of FEA has already been used in the optimisation process of a wedge-shaped TTA ceramic implant to reinforce it at the appropriate point and improve the surface topology [37]. Although the loads on the implant were also recorded, our study aimed to take a closer look at the mode and degree of effect of this operation using FEA and to evaluate it based on joint stability and load patterns.

In general, due to the naturally simulated characteristics of a computer simulation, it should be noted that the results must be interpreted in the context of this virtual representation [38]. When creating the model, assumptions from static biomechanical test setups [39,40,41] that simulate the middle of the stepping phase of the stride were adopted, which is why the applicability of the results is limited to the middle of the stepping phase of walking. Although the joint angulation of 135° was also used in cadaver studies to represent the mid-stance phase, it must be emphasised that this is variable depending on the breed [42] and that the gait pattern also changes with the occurrence of a CCLR [43] and after treatment with TTA. In addition to the joint angulation, the percentage of weight bearing on the affected limb changes significantly. In the case of an untreated CCLR, this load is almost half as high as on the contralateral side and, although it slowly equalises after treatment with TTA, it is still not at the same level 120 days later [44]. Another study reported that 90% of weight bearing was restored after TTA treatment [45]. In the virtual simulation, the vertical force was applied in even increments, but in reality, a bell-shaped curve appears, peaking in the middle of the stance phase [46]. The factors influencing joint angulation and load were deliberately ignored in order to ensure better comparability of the biomechanics and force transmission.

The musculature was limited to the gastrocnemius muscle in the simulation, and the strong involvement of the hip extensors during the lifting phase [47] was not considered. It would be beneficial to extend future research on CCL insufficiency by including these muscles to allow a more accurate simulation of real muscular activities during the lifting phase [48]. In particular, the inclusion of the hamstring group could lead to an improved understanding of the forces acting on the knee joint [49] and thus contribute to more accurate results in terms of stability and contact forces in the femorotibial joint.

The major limitation of the study is that the dynamic changes in the patellofemoral joint were not taken into account here. Therefore, the advancement of the tibia did not reach the same extent as in the cadaver or in vivo studies, as no stiffening had to be carried out in those studies. The patellofemoral joint clearly shows contact mechanical changes after the TTA, as the TTA is based on the Maquet technique [50], which originates from human medicine and reduces the patellofemoral contact forces to treat patients with painful patellofemoral osteoarthritis. Due to the representation of the ligaments and muscles as non-linear springs, performing rigid fixation in the patellofemoral joint was necessary to avoid unrealistic distortions. The simulation of ligaments and muscles as springs is a common method to represent them realistically [51,52,53], but the possibility of representing ligaments as solids [54,55] is also described and could be considered for future analyses. This allows for the implementation of models such as the Rayleigh damping model and neo-Hookean hyperelastic model, thereby enabling a more precise representation of the stress–strain curve and a more accurate depiction of the viscoelastic properties of the ligaments [56].

In this study, a constant cartilage thickness of 0.65 mm and the material properties of healthy articular cartilage were considered. However, it is relevant to note that in vivo both the cartilage thickness and the material properties are variable and depend on the extent of primary or secondary OA as well as the specific composition of proteoglycans and water in the cartilage [57]. Within this simulation, an intact medial meniscus was assumed. Therefore, our results cannot be transferred to limbs in which the medial meniscus has been removed, as is commonly performed as part of TTA treatment with meniscal injuries [12]. A retrospective study [58] of 443 diagnosed knee ligament injuries showed that such a meniscal injury was present in 36% of cases.

The subject group also showed considerable weight heterogeneity, with dogs under 20 kg, especially those around 10 kg, showing different values the most. For future studies, it is recommended that only dogs weighing 20 kg or more be included to ensure a more homogeneous database.

Joint instability responsible for the development of secondary OA also manifests itself in this model, even if only part of the total occurring advancement is simulated. The calculated advancement rates in the three conditions differed from the absolute figures of the biomechanical tests but showed the same tendency. Detailed results of the biomechanical test will be published separately in the future. The advancement of the physiological model and that of the model without CCL differed significantly. In our models, the instability inevitably appeared as cranial advancement of the tibia, as the femur was fixed in its position. However, whether in vivo it is also a cranial subluxation of the tibia or rather a caudally directed sliding movement of the femur has not yet been conclusively clarified [59]. Nonetheless, unlike in the previous cadaver studies [39,41], no significant difference was found in the model between the advancement after a CCLR and the advancement after TTA. The fact that subluxation could occur despite tibial osteotomy procedures has been previously described [9] and confirmed in vivo both in the standing position, evaluated by X-rays [60], and during the stride, evaluated by fluoroscopic video sequences [61], where subluxations persisted in 90% of the knee joints examined. Although the advancement in the present study was reduced by 19% on average, it was still 21% higher on average than with an intact CCL and thus did not differ significantly from the physiological and pathological condition.

The forces calculated in the CCL amounting to 25% of the applied force corresponded to the values of 28% calculated in another computer simulation [62] of the gait cycle at 50% of the stemming phase, whereby the maximum load of 34% occurred after 77% of the stemming phase. An unexpected finding of our study was the significant increase in forces in the PT after TTA. In addition to the increased force, there was an earlier increase in the forces, as can be seen in plots b, d and f in Figure 2 Theoretically, a 10% increase in the lever on the tibiofemoral joint was described [63], which is achieved by the distance of the attachment point of the PT and should therefore relieve the PT. Decreasing forces in the PT were observed both in cadaver studies [41,64], in which force gauges were clamped in the quadriceps, and in a multi-body dynamic simulation [65]. In the aforementioned cadaver studies, the force was applied via the femur to the tibiofemoral joint while the tibia was fixed. In contrast, in this study, the force was applied via the tibia and the femur was fixed, which may influence the effects of the force. An FEA [66], which examined the tibiofemoral effects of the Maquet procedure [50] on the human knee, showed that the force in the PT was reduced by approximately 9% in full joint extension but disappeared and even partially reversed in flexion.

In addition to these studies that support the theory, there are proven changes in the PT after TTA, similar to TPLO [67], which may indicate that a change in strain occurs after TTA. The thickening of the PT peaks immediately after surgery, but later decreases again, but the pre-operative value is no longer reached [68]. These dynamic changes could be attributed to traumatic effects during the surgical procedure. In another study [69], 50% of the 20 dogs operated on showed persistent changes in the PT, and in another study [70] with 25 dogs, all animals even showed permanent postoperative changes in the PT. A more recent study [71], which also found thickening in the PT after TTA, ruled out the possibility of the plate used in the conventional method as being the cause. The arthrotomy was omitted to minimise the possible effects of the parapatellar incision and intensive retraction of the PT, as suggested in the other studies. Despite these findings in various studies, no clear association of these changes with existing lameness or painful responses on palpation has been established to date. The clinical significance of this thickening therefore remains unclear. However, it is interesting to note that in TPLO, similar findings are found in the PT, and complications in the form of desmitis of the PT have also been described as a cause of persistent lameness [72].

The increase in forces in the PT observed in this study requires further experimental validation. The question of whether these increased forces are related to the thickening of the PT remains presumptive for now and requires further in-depth investigation. In this experimental setup, the PT bears force earlier and thus appears to counteract joint instability. There are indications that the PT may compensate for the instability by adsorbing more force, this being caused by the biomechanical changeover. As the PT was simulated as a passive structure in this model, the influence of muscle tension was not included, which could possibly lead to different results in vivo.

In this study, in addition to the forces on the PT, the loads on individual structures were also measured. The pattern observed is similar to an in vitro study by Kim et al. (2009) [39], in which the loads increased after a CCLR and returned to the initial values after TTA. With regard to implant functionality, a non-significant reduction in advancement and a reduction in peak pressures on menisci and cartilage structures were observed. The probability that an alternative implant shape, such as the classic TTA cage, would have led to different results in terms of advancement and loads appears low.

## 5. Conclusions

This FEA contains numerous simplifications, which means that direct conclusions about in vivo situations should be avoided, with fixation of the femur at the femoral head and the stiffening of the patellofemoral joint being the most significant limitations. Despite these limitations, the study provides important insights into the possibilities of in silico models, enabling an evaluation of implant behaviour to be made and providing information on joint dynamics, including the ability to measure forces on the cranial cruciate ligament a measurement that has not been feasible in in-vivo or cadaver studies. The investigated implant proves to be a promising option, as it limits the advancement and equalises the pressure conditions. At the same time, however, it causes an increased load on the patellar tendon and does not fully restore joint stability, resulting in a slight residual laxity; this aligns with in-vivo findings. One possible clinical effect of this increased load could be the observed thickening of the PT after TTA. Nevertheless, the results, and in particular the measured forces in the PT must be critically reviewed and further validated. Future FEAs of the knee joint should include the patellofemoral joint and, if possible, simulate the complete loading phase to provide a more comprehensive representation of knee joint mechanics.

## 6. Patents

A patent is pending for the ceramic implant.

## Figures and Tables

**Table 1:** Overview of material properties.

Material	Density (g/cm^3^)	Young’s Modulus (MPa)	Poisson’s Ratio	Reference
bone	2.06		0.3	Behrens et al. (2006) [26], Bartolin et al. (2021) [27]
cartilage	1	15	0.49	Koh et al. (2019) [28]
meniscus	1.1	59	0.47	Trad et al. (2018) [29]
implant	1.029	3000	0.24	Hadjicharalambous et al. (2015) [30]

**Table 2:** List of calculated forces in the patellar tendon (PT) and intact cranial cruciate ligament (CCL) (N) and the absolute figures in relation to the applied force. The columns are divided into CCL and PT, the PT is subdivided into three states (condition 1 = physiological, condition 2 = pathological, condition 3 = post-operative) and further subdivided into left and right limbs. The individual dogs are listed. In addition, the last row shows the percentage mean values ± SD of the forces in the CCL and in the PT, divided into the individual conditions.

	CCL		PT					
Condition	Condition 1		Condition 1		Condition 2		Condition 3	
Site	Left	Right	Left	Right	Left	Right	Left	Right
dog 1	39.68 23%	36.07 21%	10.2 6%	13.78 8%	30.12 18%	24.04 14%	44.5 26%	38.57 23%
dog 2	27.13 23%	32.54 28%	12.48 11%	27.65 23%	24.04 20%	40.37 23%	38.22 34%	42.42 36%
dog 3	30.12 30%	38.73 39%	6.209 6%	22.35 23%	15.28 15%	25.6 26%	31.95 32%	35 35%
dog 4	18.51 42%	- -	7.978 18%	- -	18.66 42%	- -	16.35 37%	- -
dog 5	9.09 11%	14.85 17%	0.398 0%	7.755 9%	2.072 2%	7.362 9%	3.979 5%	14.5 17%
dog 6	46.56 24%	45.66 24%	25.49 13%	38.53 20%	44.66 23%	55.57 29%	58.54 31%	60.34 32%
dog 7	15.28 36%	9.449 30%	2.635 6%	4.62 × 10^−14^ 0%	0.1069 0%	1.747 6%	4.824 11%	3.216 10%
dog 8	9.786 16%	8.333 14%	1.817 3%	4.245 7%	9.861 16%	10.26 17%	11.95 20%	11.77 19%
MV ± SD	25% ± 9.1%		10% ± 7.7%		18% ± 11.6%		25% ± 10.4%	

**Table 3:** Overview of the advancement of the tibial tuberosity (mm) in the three conditions (condition 1 = physiological, condition 2 = pathological, condition 3 = post-operative) with the percentage increase in advancement in conditions 2 and 3 compared to condition 1 and the percentage decrease in advancement (in italics, for better differentiation) in condition 3 compared to condition 2. The columns are subdivided into the three conditions and divided into left (left) and right (right) limb. The individual dogs are listed. The last row shows the mean values ± SD of the percentage increase or decrease in advance.

	Condition 1		Condition 2		Condition 3	
Site	Left	Right	Left	Right	Left	Right
dog 1	1.923	1.444	2.9 51%	2.4 66%	2.399 25% −*17%*	1.614 12% −*33%*
dog 2	1.8	1.854	2.474 37%	2.412 30%	2.117 18% −*14%*	2.129 15% −*12%*
dog 3	1.36	1.454	2.459 81%	2.98 105%	2.053 51% −*17%*	2.318 59% −*22%*
dog 4	0.743	-	1.453 96%	-	1.146 54% −*21%*	-
dog 5	1.126	1.063	1.302 16%	1.323 24%	1.084 −4% −*17%*	1.094 3% −*17%*
dog 6	2.328	2.097	3.585 54%	2.793 33%	2.271 −2% −*37%*	2.242 7% −*20%*
dog 7	0.635	0.568	1.185 87%	1.1012 78%	0.829 31% −*30%*	0.796 40% −*21%*
dog 8	0.689	0.855	1.027 49%	0.979 15%	0.892 29% −*13%*	0.85 −1% −*13%*
MV ± SD			55% ± 31.5%		21% ± 21.1% −*19% ± 7.5%*	

## Data Availability

The datasets presented in this article are not readily available due to technical limitations. Requests to access the datasets should be directed to mark.leon.lang@tiho-hannover.de.

## Appendix A

**Table A1:** Overview of the Pearson’s correlation coefficient, including *p*-values for the cranial cruciate ligament (CCL) and the patellar tendon (PT) in connection with the advancement of the tibial tuberosity and body weight (BW), divided into the three conditions (condition 1 = physiological, condition 2 = pathological, condition 3 = post-operative).

	CCL	PT		
Condition	Condition 1	Condition 1	Condition 2	Condition 3
BW—Pearson’s correlation coefficient	0.87436 <0.0001	0.73722 0.0017	0.83667 0.0001	0.80143 0.0003
advancement—Pearson’s correlation coefficient	0.88952 <0.0001	0.80453 0.0003	0.82717 0.0001	0.91273 <0.0001
